# Naphthaleneoxypropargyl-Containing Piperazine as a Regulator of Effector Immune Cell Populations upon an Aseptic Inflammation

**DOI:** 10.3390/molecules28207023

**Published:** 2023-10-11

**Authors:** Valentina K. Yu, Yelena S. Sycheva, Gulgul K. Kairanbayeva, Valery M. Dembitsky, Marina K. Balabekova, Aliya N. Tokusheva, Tulegen M. Seilkhanov, Tolganay Y. Zharkynbek, Anar Kh. Balapanova, Khaidar S. Tassibekov

**Affiliations:** 1 A.B. Bekturov Institute of Chemical Sciences, 106 Sh. Ualikhanov St., Almaty 050010, Kazakhstan; yelena-sycheva@yandex.kz (Y.S.S.); tolganay.zharkynbek@gmail.com (T.Y.Z.); kh.tassibekov@ihn.kz (K.S.T.); 2 Pathological Physiology Department, Asfendiyarov Kazakh National Medical University, 94 Tole-bi St., Almaty 050000, Kazakhstan; kairanbayeva.g@kaznmu.kz (G.K.K.); balabekova.m@kaznmu.kz (M.K.B.); aliyatokusheva88@gmail.com (A.N.T.); balapanova.an@kaznmu.kz (A.K.B.); 3 Centre for Applied Research, Innovation and Entrepreneurship, Lethbridge College, 3000 College Drive South, Lethbridge, AB T1K 1L6, Canada; 4 Laboratory of Engineering Profile NMR Spectroscopy, Sh. Ualikhanov Kokshetau State University, 76 Abai St., Kokshetau 020000, Kazakhstan; tseilkhanov@mail.ru

**Keywords:** 1-[1-(2,5-dimethoxyphenyl)-4-(naphthalene-1-yloxy)but-2-ynyl]-4-methylpiperazine, β-cyclodextrin, immune cell populations, aseptic inflammatiofn, heavy metal exposure, cadmium chloride (CdCl_2_), lead acetate (Pb(CH_3_COO)_2_)

## Abstract

This study investigated the effects of aseptic inflammation and heavy metal exposure on immune responses, as well as the potential immunomodulatory properties of the newly synthesized 1-[1-(2,5-dimethoxyphenyl)-4-(naphthalene-1-yloxy)but-2-ynyl]-4-methylpiperazine complexed with β-cyclodextrin (β-CD). Aseptic inflammation was induced by a subcutaneous injection of turpentine in rats, while heavy metal exposure was achieved through a daily administration of cadmium chloride and lead acetate. The levels of immune cell populations, including cytotoxic T lymphocytes (CTL), monocytes, and granulocytes, were assessed in the spleen. The results showed that aseptic inflammation led to decreased levels of CTL, monocytes, and granulocytes on the 14th day, indicating an inflammatory response accompanied by a migration of effector cells to the inflamed tissues. The exposure to cadmium chloride and lead acetate resulted in systemic immunotoxic effects, with reduced levels of B cells, CD4^+^ Th cells, monocytes, and granulocytes in the spleen. Notably, piperazine complexed with β-CD (the ***complex***) exhibited significant stimulatory effects on CD4^+^, CD8^+^, and myeloid cell populations during aseptic inflammation, even in the presence of heavy metal exposure. These findings suggest the potential immunomodulatory properties of the ***complex*** in the context of aseptic inflammation and heavy metal exposure.

## 1. Introduction

Previously, inflammation was considered to be a passive process, and the mechanisms underlying its resolution were not well understood. However, it is now known that inflammation plays a vital role in maintaining health as a defense mechanism. Unfortunately, in the case of diseases, the suppression of one or several immune defense components is often observed, resulting in a violation of the normal course of the inflammatory process and, in severe cases, even the death of the organism [1,2].

Studies [3,4,5] have demonstrated that inflammation resolution is an active and regulated cellular and humoral process. Impaired regulation can significantly alter the course of inflammation, transforming an acute inflammatory response into a chronic one. Certain toxic substances, industrial and environmental pollutants, as well as medications, can not only cause structural damage but also induce functional changes in immune cells. These xenobiotics can alter the differentiation ability of immune cells, disturb the expression or restriction of the major histocompatibility complex, and weaken the capacity of plasma cells to produce antibodies, particularly IgM and IgG.

Cadmium and lead are prevalent heavy metals that contaminate the environment. It has been demonstrated that these metals can modulate both innate and adaptive immune responses at different stages [6,7,8]. Specifically, they can alter the numbers of circulating B- and T-lymphocytes, NK cells and immunological memory cells, as well as the production of cytokines [6,7,8]. Lead and cadmium have been shown to stimulate the production of IgE immunoglobulins [9]. Moreover, these metals can polarize the immune response towards Th2 cells [10,11], indicating the high sensitivity of CD4^+^ Th cells to the immunotoxic effects of lead and cadmium [12,13,14,15].

Numerous molecular mediators of inflammation, including cytokines and chemokines, have been identified. These mediators play a role in attenuating the anti-inflammatory effects and facilitating the resolution of inflammation [16]. 

Recent studies on the suppressive activity of myeloid-derived suppressor cells (MDSCs) have contributed to our understanding of immune regulation disorders [17,18,19]. Despite significant progress in the investigation of the toxic effects of xenobiotics, our understanding of the impact of cadmium and lead on the inflammatory process and immune response regulation mechanisms remains limited. Additionally, there is a lack of correlational data on the effects of cadmium and lead on the activity of myeloid cells.

This study aims to address two objectives: (1) study the course of aseptic inflammation (SI) in rats exposed to cadmium chloride (CdCl_2_) and lead acetate (Pb(CH_3_COO)_2_); and (2) assess the ability of 1-[1-(2,5-dimethoxyphenyl)-4-(naphthalene-1-yloxy)but-2-ynyl]-4-methylpiperazine, in complex with β-CD (the ***complex***), to modulate the immune status of animals with SI exposed to CdCl_2_ and Pb(CH_3_COO)_2_.

## 2. Results and Discussion

Naphthol derivatives belong to an important class of chemical compounds found in both synthetic and natural products, exhibiting diverse biological activities [20,21]. Substituted naphthoxyamines have emerged as significant pharmacophores with a wide range of pharmacological and biological properties, including antitumor [22], antimicrobial [23], antibacterial [24], antiproliferative [25], antiparasitic [26], and other activities. Moreover, the search for substances that can modulate the immune status of animals with aseptic inflammation induced by heavy metal salts has been driven by the discovery of the unique properties of piperazine [27] or piperidine [28] propargyl compounds.

**Scheme 1:**
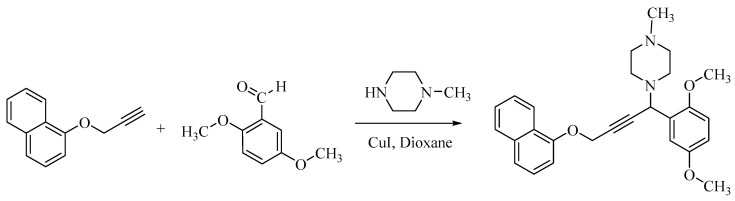
Synthesis of new 1-[1-(2,5-dimethoxyphenyl)-4-(naphthalene-1-yloxy)but-2-yn-1-yl]-4- methylpiperazine.

In this study, we synthesized a new 4-naphthoxybutynylamine derivative through the aminomethylation of 1-(prop-2-ynyloxy)naphthalene with 2,5-dimethoxybenzaldehyde and 1-methylpiperazine (Scheme 1). The reaction was conducted in absolute dioxane with catalytic amounts of copper(I) iodide at a temperature of 35–40 °C. As a result, a new 1-[1-(2,5-dimethoxyphenyl)-4-(naphthalene-1-yloxy)but-2-yn-1-yl]-4-methylpiperazine chiral mixture was obtained with a yield of 74% after its isolation from the reaction mixture. This compound structure was identified through elemental analyses, IR and NMR (^1^H and ^13^C) spectroscopy.

In the ^1^H NMR spectra (Figure S2) of the 4-naphthoxybutynylamine derivative, characteristic proton signals are observed. The aminomethine group shows signals at 4.95 ppm. The methylene groups of the piperazine ring exhibit signals at 2.34 and 2.56 ppm. The methyl group attached to the nitrogen of the diazaheterocycle appears at 2.15 ppm. Proton signals from the O-CH_3_ group are observed at 3.39 and 3.63 ppm, while the protons of the O-methylene group resonate at 4.86 ppm. The aromatic protons resonate in the downfield region (6.66–8.21 ppm).

In the ^13^C NMR spectra (Figure S3) of the 4-naphthoxybutynylamine derivative, the signal of the carbon atom in the aminomethine group is observed at 54.91 ppm. The CH_3_–N group exhibits a signal at 45.89 ppm. The carbon atoms of the piperazine ring resonate at 49.44 and 54.44 ppm. The carbon atoms of the O–CH_3_ group are registered at 56.51 and 56.74 ppm. The carbon atoms of the triple bond (C≡C) appear at 81.24 and 85.24 ppm. A signal at 54.37 ppm is assigned to the oxymethylene carbon. The carbon atoms of the aromatic rings show signals in the weak field region (105.77–153.44 ppm).

A structure of the new 4-naphthoxybutynylamine derivative has been confirmed with 2D NMR spectroscopy of COSY (^1^H–^1^H), HMQC (^1^H–^13^C) and HMBC (^1^H–^13^C). Thus, a homo- and hetero-nuclear spin–spin coupling has been established (Figures S4–S6, Supporting Information).

**Figure 1:**
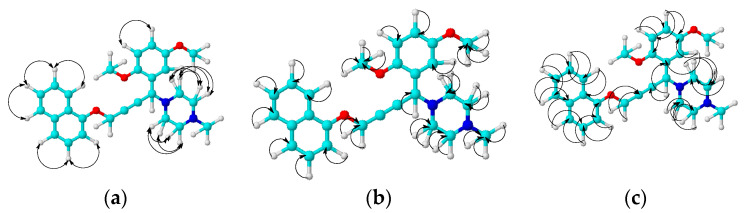
The structural correlations COSY (**a**), HMQC (**b**) and HMBC (**c**) in NMR spectra of new 4-naphthoxybutynylamine derivative.

The observed NMR correlations of COSY (^1^H–^1^H), HMQC (^1^H–^13^C) and HMBC (^1^H–^13^C) in a molecule of the 4-naphthoxybutynylamine derivative are illustrated in Figure 1.

The COSY (^1^H–^1^H) spectra of the molecule demonstrate the spin–spin correlations through proton bonds. The hetero-nuclear coupling of protons with carbon atoms through a single bond have been established by HMQC (^1^H–^13^C) spectroscopy. A hetero-nuclear coupling of a proton with carbon atoms through two or more bonds has been determined by HMBC (^1^H–^13^C) spectroscopy. 

The oily 1-[1-(2,5-dimethoxyphenyl)-4-(naphthalene-1-yloxy)but-2-yn-1-yl]- 4-methylpiperazine was complexed with β-CD using the standard procedure [29].

The inclusion complex of 1-[1-(2,5-dimethoxyphenyl)-4-(naphthalene-1-yloxy) but-2-ynyl]-4-methylpiperazine with β-CD is formed by the interaction of an ethanol solution of 1-[1-(2,5-dimethoxyphenyl)-4-(naphthalene-1-yloxy)but-2-ynyl]- 4-methylpiperazine with an aqueous solution of β-CD, taken in a mass ratio of 1:1, at a reaction temperature of 45 °C, the reaction is carried out for 5 h. The progress of the reactions was monitored using thin-layer chromatography on silica gel until the disappearance of the starting product (R_f_ 0.70) in an aqueous-alcohol solution. The product was collected after the slow evaporation of the solution by forming a powder product. The complex was identified through elemental analysis and IR spectroscopy.

A comparative analysis of the IR spectra (Figures S1 and S7–S9) of 1-[1-(2,5-dimethoxyphenyl)-4-(naphthalene-1-yloxy)but-2-ynyl]-4-methylpiperazine (*guest*), β-cyclodextrin (*host*) and the ***complex*** showed the identity of the spectra (Figures S7 and S8) of the last two products, indicating the complete entry of the *guest* molecule into the *host* cavity. Grinding the 4-naphthoxybutynylamine derivative with β-CD in a 1:1 ratio in an agate mortar also led to the formation of a complex (Figure S9). 

Previously, we conducted a study on the effect of vanadium and chromium on inflammatory processes. As a result of our research, it was found that metals have a negative effect on the development of inflammatory processes and slow down the resolution process. Microscopic studies have established that at the initial stage, there is a decrease in the infiltration of neutrophils and monocytes into the inflammation site. This decrease in immune cell activity contributes to the formation of areas of necrosis, which are more noticeable than in the animals that were not administered vanadium and chromium compounds (SI group). Such results indicate the presence of a mild inflammatory phase and cause a delay in the transition to the reparative phase of inflammation [18].

To evaluate the impact of cadmium chloride and lead acetate on the progression of aseptic inflammation and assess the ability of the ***complex*** to modulate the immune status of animals exposed to heavy metal salts in the SI group, a histological analysis was performed. 

To obtain the most objective assessment of the severity of sterile inflammation and its characteristics in different groups of animals, we conducted a semi-quantitative assessment of the morphogenesis of aseptic wounds [30] considering the following parameters:

1. The size of necrosis: the extent of the area affected by tissue necrosis.
2. Soft-tissue swelling: measuring the degree and severity of swelling around the site of inflammation.
3. The focus of infiltration by neutrophils, monocytes and eosinophils: the determination of the number of typical inflammatory cells penetrating the inflammatory zone.
4. The intensity of vascular proliferation: the degree of activity in the formation of new vessels in the area of inflammation.
5. The formation of granulation tissue: the effectiveness of the process of formation of granulation tissue necessary for the regeneration of damaged tissues.

**Figure 2:**
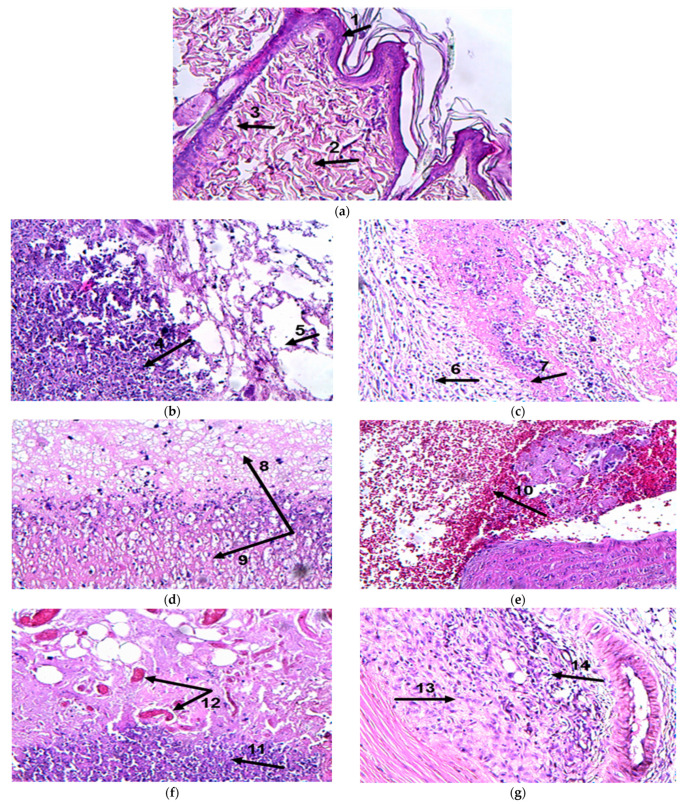
Histological images at the original ×200 magnification of the 7 independent experiments for group C (**a**); the SI groups on the 7th day (**b**) and the 14th day (**c**); the Me/SI groups on the 7th day (**d**) and the 14th day (**e**) and the Me/SI/***complex*** groups on the 7th day (**f**) and the 14th day (**g**).

A blind semi-quantitative method was employed in all experimental groups using a scoring system from 0 to 3 (0—no pathology, 1—weak, 2—moderate, 3—severe pathology). Please refer to Table 1 and Figure 2 for the results.

Figure 2a (C) 1—epidermis, 2—subepithelial connective tissue, 3—hair follicle; Figure 2b (SI, the 7th day) 4—a leukocyte infiltration, consisting of a small amount of monocytes, histiocytes and eosinophilic leukocytes, 5—edema; Figure 2c (SI, the 14th day) 6—the mature granulation tissue with the formation of a connective tissue capsule, 7—decrease in the area of the inflammation; Figure 2d (Me/SI, the 7th day) 8—a wide area of purulent-necrotic inflammation, 9—resorption of purulent-necrotic detritus by macrophages; Figure 2e (Me/SI, the 14th day) 10—an extensive hemorrhage; Figure 2f (Me/SI/complex, the 7th day) 11—a zone of inflammation with an abundance of macrophages, 12—vascular plethora; Figure 2g (Me/SI/complex, the 14th day) 13—a proliferation of the loose fibrous connective tissue, 14—a single perifocal lymphohistiocytic infiltration. 

The use of the ***complex*** improves the wound healing process due to immune modulation, the growth of granulation tissue and the regeneration of blood vessels.

The tissues with aseptic inflammation from the SI and Me/SI groups exhibited significantly higher pathological changes compared to group C (*p* < 0.01). On the 7th and 14th days after the development of inflammation, the Me/SI group showed a pronounced necrotic focus, soft tissue edema, and the absence of small vessel proliferation compared to the SI rats (*p* < 0.01). The administration of the ***complex*** significantly improved the indicators of necrosis, edema, vessel proliferation and fibroblast activity in both the SI and Me/SI groups (*p* < 0.01).

**Figure 3:**
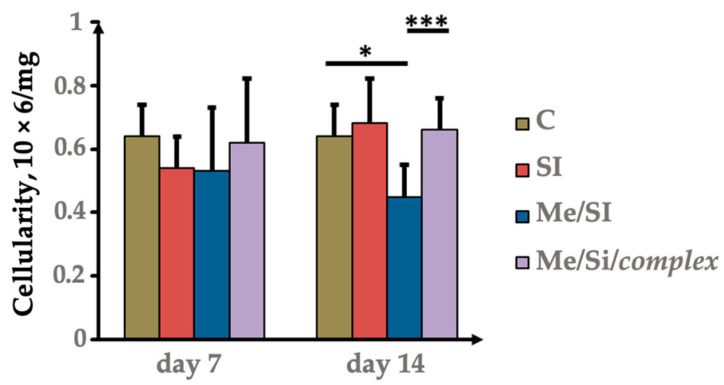
Cellularity of the spleen (×10^6^ cells/mg) in the control and experimental groups of animals in the dynamics of observation. The calculated *p*-values are given as: * *p <* 0.05 compared to C; *** *p* < 0.05 compared to corresponding Me/SI group.

The immunotoxic effect of CdCl_2_ and Pb(CH_3_COO)_2_ was manifested by a violation of the cellularity of the spleen of the experimental animals. The corresponding results of assessing the dynamics of changes in spleen cellularity and statistical data on the significance of differences between the experimental groups are presented in Figure 3.

The summarized data are presented as M ± SD. In groups C, SI, Me/SI and Me/SI/***complex***, spleen cellularity was assessed. To calculate the cellularity of the spleen, the cell concentration was multiplied by the volume of the cell suspension and divided by the mass of the lymph organ. 

The analysis performed revealed a statistically significant decrease in spleen cellularity in the Me/SI group on the 14th day to 0.4 ± 0.1 (*p* = 0.0046) compared with the control group (0.6 ± 0.1). However, as can be seen from Figure 3, the introduction of the ***complex*** led to an increase in spleen cellularity in the Me/SI/***complex*** group up to 0.7 ± 0.1 on the 14th day compared to the Me/SI group (0.4 ± 0.1, *p* = 0.0064), which, apparently, was associated with the immunostimulating effect of the ***complex*** on splenocytes.

When assessing the main effector populations of T cells, it was found that on the 14th day of observation, there was a twofold decrease in the CD4^+^ Th cell content in the Me/SI group compared to group C (18.4 ± 3.9% on the 7th day and 15.2 ± 3.6% on the 14th day, *p* = 0.003 and *p* = 0.001, respectively), as well as compared to the SI group (25.5 ± 3.9% on the 7th day and 26.5 ± 5.9% on the 14th day, *p* = 0.001 and *p* = 0.00007, respectively).

The Th cells are highly susceptible to the toxic effects of cadmium and lead among all lymphocyte populations [2]. The administration of the ***complex*** significantly increased the level of CD4^+^ Th cells in the Me/SI/***complex*** group by 35.2 ± 6.5% (on the 7th day) and 31.4 ± 4.5% (on the 14th day) compared to the Me/SI group (*p* = 0.002 and *p* = 0.0001, respectively), reaching values comparable to the control group.

**Figure 4:**
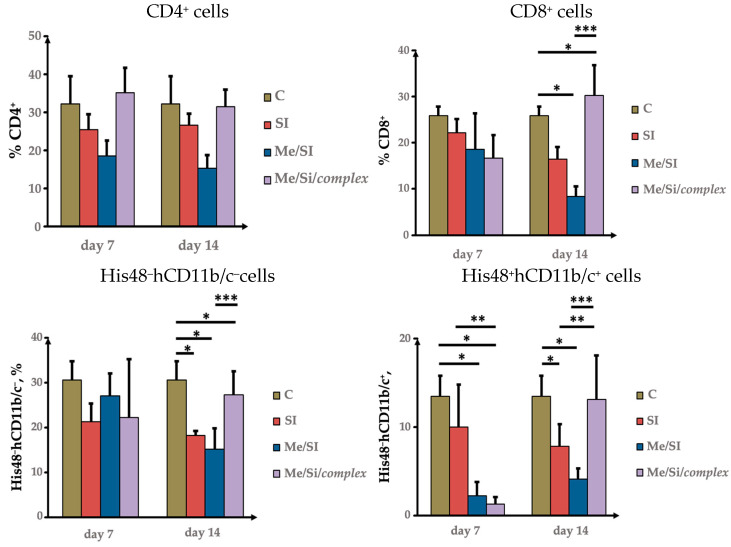
The proportion of CD4^+^, CD8^+^, His48^+^hCD11b/c^+^, His48^−^hCD11b/c^+^ cells in the observation dynamics. The *p*-values are indicated as follows: * *p* < 0.05 compared to C; ** *p* < 0.05 compared to the corresponding SI group; *** *p* < 0.05 compared to the corresponding Me/SI group.

An analysis of T-lymphocytes revealed a gradual decrease in the CD8^+^ T-cell content during aseptic inflammation (Figure 4), along with a decrease in activated CD4^+^ Th cells expressing the IL-2 receptor CD25, by 2.1 ± 0.9% and 3.2 ± 0.8% on the 7th and 14th days, respectively, compared to group C (4.5 ± 0.7%, *p* = 0.0004 and *p* = 0.02). Interestingly, the administration of the ***complex*** significantly increased the content of cytotoxic T lymphocytes (CTL) to the level observed in the C group by the 14th day (*p* = 0.04).

The pretreatment with heavy metals resulted in a further decrease in CD8^+^ among the T cells populations in the Me/SI group compared to groups C (*p* = 0.00001) and SI (*p* = 0.0009) (Figure 4), as well as a decrease in CD4^+^25^+^-activated T cells on the 14th day compared to group C (*p* = 0.002). The administration of the ***complex*** significantly increased CTL levels to the level observed in the C group on the 14th day compared to the Me/SI group (*p* = 0.002).

The ***Complex*** modulated the splenocytes in the spleen of rats with cadmium- and lead-induced aseptic inflammation. The data are presented as M ± SD. Flow cytometry was used to analyze the levels of CD4^+^, CD8^+^, His48^+^hCD11b/c, and His48^–^hCD11b/c in the C, SI, Me/SI, and Me/SI/***complex*** groups. Monoclonal antibodies specific to surface markers were used according to the manufacturer’s protocols (Figure 4). 

Neutrophils and monocytes/macrophages play a crucial role in the inflammatory process [13]. Hence, we further investigated the impact of heavy metal salts on the levels of neutrophils (His48^–^hCD11b/c^+^) and monocytes (His48^+^CD11b/c^+^) within the cell populations [14]. In the experimental groups of the SI and Me/SI animals, a gradual decrease in the monocyte levels in the spleen was observed during aseptic inflammation. This decrease reached statistical significance in all groups compared to the C group by the 14th day following turpentine administration. This pattern can be attributed to the migration of monocytes from circulation into the tissues at the site of inflammation. Notably, the ***complex*** significantly increased the number of cells with the His 48^–^CD11b/c^+^ phenotype in the Me/SI/***complex*** group compared to the Me/SI group (*p* = 0.001) on the 14th day of observation.

When assessing the granulocytic cell population over time, we observed a low level of neutrophils in the spleen of the SI group by the 14th day compared to the C group (*p* = 0.006).

A similar decrease in granulocytes compared to the C group was observed in the Me/SI group on the 14th day (*p* = 0.009). In the animals pretreated with cadmium and lead compounds, the inflammation was accompanied by a more significant decrease in the proportion of His48^–^hCD11b/c^+^ cells on the 7th and 14th days compared to the SI and C groups. The administration of the ***complex*** significantly increased the content of His48^+^hCD11b/c^+^ cells in the Me/SI/***complex*** group, comparable to the levels observed in the SI and Me/SI groups, respectively, and similar to the levels in the C group. This suggests that the observed increase in spleen cellularity in the Me/SI/***complex*** group on the 14th day compared to the 7th day may be attributed to the specific effect of the ***complex*** on granulocytes.

## 3. Materials and Methods

### 3.1. Chemical Experimental Part

#### 3.1.1. Methods of Synthesis and Structure Studies for the Chemical Compound

The progress of the reactions and the purity of the products were monitored using a TLC (thin-layer chromatography) analysis on “Silufol UV-254” plates, visualized by the appearance of substance spots with iodine vapor. The eluent used for TLC was a benzene–ethanol mixture (3:1). An elemental analysis was performed using a Rapid Micro N Cube elemental analyzer. IR spectra were recorded using a Thermo Scientific Nicolet 5700 FTIR spectrometer with KBr pellets. The ^1^H and ^13^C NMR spectra of the samples were recorded on a JNM-ECA 400 (Jeol) spectrometer operating at frequencies of 399.78 MHz (^1^H) and 100.53 MHz (^13^C) in deuterated chloroform (CDCl_3_).

#### 3.1.2. Synthesis of 1-(1-(2,5-Dimethoxyphenyl)-4-(naphthalene-1-yloxy)but-2-yn-1-yl)- 4-methylpiperazine

The synthesis of 1-(1-(2,5-dimethoxyphenyl)-4-(naphthalene-1-yloxy)but-2-yn-1-yl)- 4-methylpiperazine was performed by reacting 2.0 g (0.0109 mol) of 1-(prop-2-ynyloxy)-naphthalene, 1.82 g (0.0109 mol) of 2,5-dimethoxybenzaldehyde and 1.09 g (0.0109 mol) of 1-methylpiperazine in the presence of 0.2 g of copper(I) iodide in 20 mL dioxane at 40 °C for 2h, and then 3.48 g (74%) of synthesized product was obtained as an oil after removing the dioxane solution from the reaction mixture using a rotary evaporator. Its R_f_ value was 0.70 (benzene–ethanol, 3:1).

The results of the elemental analysis for C_27_H_30_N_2_O_3_ are as follows: C, 75.32%; H, 7.02%; N, 6.51%; and O, 11.15%. The values obtained are C, 75.47%; H, 7.17%; and N, 6.67%.

IR (KBr, ν, cm^−1^): 1698, 1586, 1486, 1398, 1265, 1031, 910, 885, 726.

^1^H NMR (CDCl_3_, δ, ppm): 2.15 (s, 3H, N–CH_3_), 2.34 (s, 4H, N(CH_2_CH_2_)_2_N–CH_3_), 2.56 (m, 4H, N(CH_2_CH_2_)_2_N–CH3), 3.39 (m, 3H, OCH_3_), 3.63 (s, 3H, OCH_3_), 4.86 (s, 2H, OCH_2_), 4.95 (s 1H, CH–N), 6.66 (s, 2H, ArH), 6.89–6.98 (m, 2H, PhH), 7.26–7.37 (m, 4H, ArH), 7.69 (s, 1H, ArH), 8.21 (s, 1H, ArH).

^13^C NMR (CDCl_3_, δ, ppm): 45.89 (N–CH_3_), 49.44, 55.44 (N(CH_2_)_2_), 56.51, 56,74 (O–CH_3_), 54.37 (O–CH_2_), 54.91 (N–CH), 81.24, 85.24 (C≡C), 105.77, 112.81, 113.62, 115.84, 120.89, 123.33, 125.79, 126.62, 126.99, 127.43, 134.57, 151.31, 153.44 (ArC).

^1^H–^1^H COSY: H^10^–H^11^ (6.66, 6.93; 6.93, 6.66), H^24^–H^23^ (6.86, 7.25; 7.25, 6.86), H^26^–H^27^(7.36, 7.66; 7.66, 7.36), H^26^–H^25^ (7.35, 8.19; 8.19, 7.35). ^1^H–^13^CHMQC: H^14^–C^14^ (2.13, 46.15), H^2ax,6ax,2eq,6eq^-C^2,6^ (2.54, 49.39), H^3ax,5ax^-C^3,5^(2.31, 55.15), H^30^–C^30^ (3.61, 57.13), H^32^–C^32^ (3.41, 55.75), H^3eq,5eq^-C^3,5^ (3.40, 55.69), H^7^–C^7^ (4.93, 54.25), H^17^–C^17^ (4.84, 56.59), H^28^–C^28^ (7.36, 126.73), H^26^–C^26^ (7.36, 125.29), H^10^–C^10^ (6.96, 115.98), H^13^–C^13^ (6.96, 115.98), H^11^–C^11^ (6.64, 112.62), H^24^–C^24^ (6.85, 105.69), H^22^–C^22^ (7.32, 120.88), H^27^–C^27^ (7.65, 127.63); H^25^–C^25^ (8.18, 122.24).

#### 3.1.3. The Inclusion Complex of 1-[1-(2,5-Dimethoxyphenyl)-4-(naphthalene-1-yloxy)- but-2-ynyl]-4-methylpiperazine with β-cyclodextrin (Complex)

A solution containing 1.3 g of 1-[1-(2,5-dimethoxyphenyl)- 4-(naphthalene-1-yloxy)but-2-ynyl]-4-methylpiperazine in 20 mL of ethanol and a separate solution of 1.3 g of β-CD in 20 mL of distilled water were mixed at a temperature of 45–50 °C for 5 h (reaction control was carried out by TLC until the spot disappeared with R_f_ 0.70—1-[1-(2,5-dimethoxyphenyl)-4-(naphthalene-1-yloxy)-but-2-ynyl]-4-methylpiperazine, in an aqueous-alcohol solution). As a result, 1.1 g (42%) of the complex between 1-[1-(2,5-dimethoxyphenyl)-4-(naphthalene-1-yloxy)-but-2-ynyl]-4-methylpiperazine and β-CD was obtained. The complex appeared as a light yellow powder, melting with decomposition above 240 °C.

The results for the elemental analysis for C_69_H_100_N_2_O_38_ are as follows: C, 52.94%; H, 6.44%; N, 1.79%; and O, 38.84%. The values obtained are C, 52.83%; H, 6.31%; and N, 1.67%.


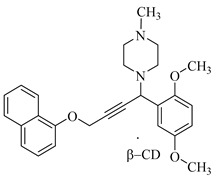


### 3.2. Biological Experimental Part

The studies were conducted using an acoustic focusing cytometer Attune™ NxT (Thermo Fisher Scientific, Waltham, MA, USA, manufacturer country – Life Technologies holdings PTE LTD, CRN/UEN: 200104491K Marsiling Industrial Estate Road 3 # 07-06, Singapore, Singapore). 

The following reagents were used during the experiments: phosphate-buffered saline solution (PBS) (Sigma-Aldrich, Milwaukee, WI, Germany), FACS Lysing Solution for erythrocyte lysis, Cyto Fix Fixation Buffer (BD Biosciences, Mountain View, CA, USA), APC-labeled anti-rat CD3 (0.1 mg, BD Biosciences, Mountain View, CA, USA), Cy-Chrome™-labeled anti-rat CD4 (0.1 mg, BD Biosciences, Mountain View, CA, USA), PerCP-labeled anti-rat CD8a (0.1 mg, BD Biosciences, Mountain View, CA, USA), PE-labeled anti-rat CD1lb/c (0.2 mg, BD Biosciences, Mountain View, CA, USA), FITC-labeled anti-rat Granulocytes (0.5 mg, BD Biosciences, Mountain View, CA, USA), FITC-labeled anti-His48 (BD Biosciences, Mountain View, CA, USA), FITC labeled anti-rat CD4 (0.5 mg, BD Biosciences, Mountain View, CA, USA). 

The preparation and staining of splenocytes for flow cytometry: After isolating the spleen cells, the supernatant fluid is removed from the resulting mixture as much as possible. Subsequently, erythrocyte lysis is carried out using 2 mL of high-yield lyse solution. Centrifugation is carried out at 500 RCF for 5 minutes with the addition of 2 mL of 0.9% saline. A mixture of antibodies (APC-labeled anti-CD3, PE-Cy5-labeled anti-CD4, PerCP-labeled anti-CD8a, FITC-labeled anti-His48, PE-labeled anti-CD11b/c, PE-labeled anti-IL-4, and FITC-labeled anti-IFNc) with 0.9% physiological solution is added to the prepared cell suspension, mixed well and incubated for 30 minutes at +4 degrees in a refrigerator. Cell fixation/permeabilization solution is prepared from fixation/permeabilization concentrate and fixation/permeabilization diluent and incubated for 30 minutes at room temperature in the dark (following by order). The results are analyzed on a flow cytometer. 

Biotesting: 42 outbred male albino rats weighing 180–220 g were used with a spread within ±20% of the average weight [18,30]. The animals were kept under standard vivarium conditions at room temperature, 22.5–23.0 °C, and had free access to food and water. The experiments were carried out in accordance with the International Guidelines for Biomedical Research in Animals developed by CIOMS and ICLAS (2012) [31]. Study approval was provided by the Local Ethics Committee of Asfendiyarov Kazakh National Medical University, protocol No. 4 (95) of 29 April 2020.

Experiment design: To conduct this research, the animals were randomly divided into four groups. Each group (except the control) was divided into 2 subgroups of 6 rats, and on each, studies were conducted 7 and 14 days after the start of modeling sterile inflammation. The first group (C) consisted of intact animals that were not exposed to any experimental treatments, serving as the control group. The second group (SI) underwent sterile inflammation modeling. A subcutaneous injection of 0.3 mL of turpentine oil was carried out after treatment with 70% ethanol [32].

The third group (Me/SI) was subjected to a turpentine injection after a two-week course of administering heavy metal salt solutions. Combined seed with cadmium chloride and lead acetate was administered at a dose of 2.5 mg/kg and previously dissolved in phosphate–salt buffer. The resulting solutions were administered orally through an esophageal catheter daily for two weeks. Both the C and SI groups received an equal volume of PBS orally.

One group within the Me/SI category received the ***complex*** (Me/SI/***complex***) in a volume of 325 mg/kg of body weight following the turpentine injections for a period of 10 days. They were euthanized by rapid decapitation, with Zoletil-Xylazine anesthesia used additionally for a painless beheading [33].

In each series of experiments, on the 7th and 14th days after the last oral administration of CdCl_2_ and Pb(CH_3_COO)_2_ and the beginning of modeling of sterile inflammation, an autopsy was performed, after which the spleen (N6) was surgically removed. Immunological studies of the subpopulations of myeloid suppressor cells were carried out in rat splenocytes: the assessment included the evaluation of the main T-lymphocyte populations (CD4^+^ (Th), CD8a^+^ (CTL)) and the proportion of myeloid cells (CD11b/c^+^His48^−^, CD11b/c^+^His48^+^, CD11b/c^+^His48 low, CD11b/c^+^His48 high) in each group.

Histopathological evaluation: For the morphological studies at a slaughter, the inflammatory tissue was collected, which was fixed in 10% neutral formalin or Carnoy’s fixative. After the fixation, the inflammation tissues were washed from the fixative, dehydrated, and embedded in paraffin. After the standard posting of the material, paraffin sections 7–8 mm thick were stained with hematoxylin-eosin [34]. The preparations were examined using an Axio ZEISS Lab.A1 light microscope with a built-in AxioCamERc5s digital camera (Germany) at magnifications of 100 and 200. During the microscopic examination of the material, special attention was paid to the histomorphological picture of the inflammatory tissue of the experimental individuals, and a comparative assessment was carried out.

Statistical analysis: The obtained experimental parameters were analyzed using one-way ANOVA analysis with the post hoc Tukey–Kramer test, and a value of *p* < 0.05 was considered statistically significant. The values were expressed as the mean ± standard deviation of at least six independent experiments. GraphPadPrism 4 was used to create and design the graphics of the data.

## 4. Conclusions

In this study, it has been demonstrated that during aseptic inflammation induced by the subcutaneous injection of 0.3 mL of turpentine, a decrease in the levels of cytotoxic T lymphocytes (CTL), monocytes, and granulocytes occurs by the 14th day of observation. The inflammatory response to turpentine administration is associated with the migration of effector cells from circulation to the inflamed tissues, accompanied by a decrease in immunosuppressive factors.

Furthermore, the daily administration of cadmium chloride and lead acetate to rats for two weeks resulted in a systemic immunotoxic effect, manifested by decreased spleen cellularity and levels of B cells, CD4^+^ Th cells, monocytes, and granulocytes in the spleen.

Interestingly, the ***complex*** (1-[1-(2,5-dimethoxyphenyl)-4-(naphthalene-1-yloxy)- but-2-ynyl]-4-methylpiperazine as a complex with β-cyclodextrin) demonstrated significant stimulatory effects on the populations of the CD4^+^, CD8^+^, and myeloid cells during aseptic inflammation, even in the presence of heavy metal exposure.

These findings suggest that the ***complex*** has potential immunomodulatory properties in the context of aseptic inflammation and heavy metal exposure. Further research is warranted to explore its therapeutic applications and mechanisms of action.

## Figures and Tables

**Table 1:** The characteristic changes in the skin tissues of the C, SI, Me/SI and Me/SI/complex animals 7 and 14 days after the development of aseptic inflammation.

The Scores in Groups (for Each Group, n = 6)	Parameters			
	Necrosis	Edema	Neutrophils, Monocytes, Eosinophils	Angiogenesis, Connective Tissue
	(Minimum–Maximum)			
	Day 7			
C	(0–0)	(0–0)	(0–0)	(0–0)
SI	(1–3) *	(1–3) *	(1–3) *	(0–2) *
Me/SI	(3–3) *+	(2–3) *+	(1–3) *	(0–0) +
Me/SI/***complex***	(0–1) +^	(0–1) +^	(2–3) *	(0–1) ^
	Day 14			
C	(0–0) *	(0–0) *	(0–0) *	(0–0) *
SI	(0–2) *	(0–2) *	(0–3) *	(1–3) *
Me/SI	(2–3) *+	(2–3) *+	(2–3) *+	(0–1) +
Me/SI/***complex***	(0–1) +^	(0–0) +^	(0–1) +^	(2–3) +^

NOTE: significant differences, assessed by one-way ANOVA analysis, are defined as: * *p* < 0.01 in comparison with the C group; + *p* < 0.01 in comparison with the respective SI group. ^ *p* < 0.01 in comparison with the respective Me/SI group. The minimal–maximal pathological values in each group are placed in parentheses.

## Data Availability

The datasets used and/or analyzed during the present study are available from the corresponding author on reasonable request.

## Supplementary Materials

The following supporting information can be downloaded athttps://www.mdpi.com/article/10.3390/molecules28207023/s1: Figure S1: IR (KBr, ν, cm^−1^) spectrum of 1-[1-(2,5-dimethoxyphenyl)-4 (naphthalene-1-yloxy)but-2-ynyl]-4-methylpiperazine; Figure S2: 1H NMR (399.78 MHz, CDCl_3_) spectrum of 1-[1-(2,5-dimethoxyphenyl)-4-(naphthalene-1-yloxy)but-2-ynyl]-4-methylpiperazine; Figure S3: ^13^C NMR (100.53 MHz, CDCl_3_) spectrum of 1-[1-(2,5-dimethoxyphenyl)-4-(naphthalene-1-yloxy)but-2-ynyl]-4-methylpiperazine; Figure S4: ^1^H-^1^H COSY spectrum of 1-[1-(2,5-dimethoxyphenyl)-4-(naphthalene-1-yloxy)but-2-ynyl]-4-methylpiperazine; Figure S5: ^1^H-^13^C HMQC spectrum of 1-[1-(2,5-dimethoxyphenyl)-4-(naphthalene-1-yloxy)but-2-ynyl]-4-methylpiperazine; Figure S6: ^1^H-^13^C HMBC spectrum of 1-[1-(2,5-dimethoxyphenyl)-4-(naphthalene-1-yloxy)but-2-ynyl]-4-methylpiperazine; Figure S7: IR (KBr, ν, cm^−1^) spectrum of β-cyclodextrin (β-CD); Figure S8: IR (KBr, ν, cm^−1^) spectrum of 1-[1-(2,5-dimethoxyphenyl)-4-(naphthalene-1-yloxy)but-2-ynyl]-4-methylpiperazine with β-cyclodextrin (***complex***); Figure S9: IR (KBr, ν, cm^−1^) spectrum of 1-[1-(2,5-dimethoxyphenyl)-4-(naphthalene-1-yloxy)but-2-ynyl]-4-methylpiperazine with β-cyclodextrin (physical mixture).

## Sample Availability

Samples of the compounds are available from the authors upon reasonable request.

## References

[1] Grimm M., Feyen O., Hofmann H., Teriete P., Biegner T., Munz A., Reinert S. (2016). Immunophenotyping of patients with oral squamous cell carcinoma in peripheral blood and associated tumor tissue. Tumour Biol..

[2] Vignali D.A., Collison C.J., Workman C.J. (2008). How regulatory T cells work. Nat. Rev. Immunol..

[3] Zhou J., Chu H., Li C., Wong B.H., Cheng Z.S., Poon V.K., Sun T., Lau C.C., Wong K.K., Chan J.Y. (2014). Active replication of Middle East respiratory syndrome coronavirus and aberrant induction of inflammatory cytokines and chemokines in human macrophages: Implications for pathogenesis. J. Infect. Dis..

[4] Zhao J., Bulek K., Gulen M.F., Zepp J.A., Karagkounis G., Martin B.N., Zhou H., Yu M., Liu X., Huang E. (2015). Human Colon Tumors Express a Dominant-Negative Form of SIGIRR That Promotes Inflammation and Colitis-Associated Colon Cancer in Mice. Gastroenterology.

[5] Lai D., Qin C., Shu Q. (2014). Myeloid-Derived Suppressor Cells in Sepsis. Biomed. Res. Int..

[6] Ebrahimi M., Khalili N., Razi S., Keshavarz-Fathi M., Khalili N., Rezaei N. (2020). Effects of lead and cadmium on the immune system and cancer progression. J. Environ. Health Sci. Eng..

[7] Zhang Y., Xu X., Sun D., Cao J., Zhang Y., Huo X. (2017). Alteration of the number and percentage of innate immune cells in preschool children from an e-waste recycling area. Ecotoxicol. Environ. Saf..

[8] Lafuente A., González-Carracedol A., Esquifino A.I. (2004). Differential effects of cadmium on blood lymphocyte subsets. Biometals.

[9] Skoczyńska A., Poreba R., Sieradzki A., Andrzejak R., Sieradzka U. (2002). The impact of lead and cadmium on the immune system. Med. Pr..

[10] Heo Y., Mondal T.K., Gao D., Kasten-Jolly J., Kishikawa H., Lawrence D.A. (2007). Posttranscriptional inhibition of interferon-gamma production by lead. Toxicol. Sci..

[11] Turley A.E., Zagorski J.W., Kennedy R.C., Freeborn R.A., Bursley J.K., Edwards J.R., Rockwell C.E. (2019). Chronic low-level cadmium exposure in rats affects cytokine production by activated T cells. Toxicol. Res..

[12] Samie N., Muniandy S., Kanthimathi M.S., Haerian B.S. (2016). Mechanism of action of novel piperazine containing a toxicant against human liver cancer cells. PeerJ..

[13] Alexia C., Cren M., Louis-Plence P., Vo D.N., El Ahmadi Y., Dufourcq-Lopez E., Lu Z.Y., Hernandez J., Shamilov F., Chernysheva O. (2019). Polyoxidonium^®^ Activates Cytotoxic Lymphocyte Responses Through Dendritic Cell Maturation: Clinical Effects in Breast Cancer. Front. Immunol..

[14] Lafuente A., González-Carracedo A., Romero A., Esquifino A.I. (2003). Effect of cadmium on lymphocyte subsets distribution in thymus and spleen. J. Physiol. Biochem..

[15] Chaudhry A., Rudensky A.Y. (2013). Control of inflammation by integration of environmental cues by regulatory T cells. J. Clin. Investig..

[16] Robb C.T., Regan K.H., Dorvar D.A., Rossi A.G. (2016). Key mechanisms regulating the resolution of lung inflammation. Semin. Immunopathol..

[17] Zhang W., Fang X., Gao C., Song C., He Y., Zhou T., Yang X., Shang Y., Xu J. (2023). MDSCs in sepsis-induced immunosuppression and its potential therapeutic targets. Cytokine Growth Factor Rev..

[18] Balabekova M.K., Ostapchuk Y.O., Perfilyeva Y.V., Tokusheva A.N., Nurmuhambetov A., Tuhvatshin R.R., Trubachev V.V., Akhmetov Z.B., Abdolla N., Kairanbayeva G.K. (2021). Oral administration of ammonium metavanadate and potassium dichromate distorts the inflammatory reaction induced by turpentine oil injection in male rats. Drug. Chem. Toxicol..

[19] Van Geffen C., Heiss C., Deißler A., Kolahian S. (2022). Pharmacological modulation of myeloid-derived suppressor cells to dampen inflammation. Front. Immunol..

[20] Vargas J.A.M., Day D.P., Burtoloso A.C.B. (2021). Substituted Naphthols: Preparations, Applications, and Reactions. Eur. J. Org. Chem..

[21] Makar S., Saha T., Singh S.K. (2019). Naphthalene, a versatile platform in medicinal chemistry: Sky-high perspective. Eur. J. Med. Chem..

[22] Wang G., Liu W., Tang J., Ma X., Gong Z., Huang Y., Li Y., Peng Z. (2020). Design, synthesis, and anticancer evaluation of benzophenone derivatives bearing naphthalene moiety as novel tubulin polymerization inhibitors. Bioorg. Chem..

[23] Kottapalle G., Shinde A. (2021). Synthesis of 1-(2-substitutedphenyl-2,3-dihydro-1*H*-benzo[*b*][1,4]diazepin-4-yl)naphthalene-2-ol under different solvent conditions as a potent antimicrobial agent. Chem. Data Collections.

[24] Roman G., Năstasă V., Bostănaru A.C., Mareş M. (2016). Antibacterial activity of Mannich bases derived from 2-naphthols, aromatic aldehydes and secondary aliphatic amines. Bioor. Med. Chem. Lett..

[25] Cherfaoui B., Guo T.K., Sun H.P., Cheng W.L., Liu F., Jiang F., Xu X.L., You Q.D. (2016). Synthesis and evaluation of 4-(2-hydroxypropyl)piperazin-1-yl) derivatives as Hsp90 inhibitors. Bioor. Med. Chem..

[26] Zuffo M., Stucchi A., Campos-Salinas J., Cabello-Donayre M., Martínez-García M., Belmonte-Reche E., Pérez-Victoria J.M., Mergny J.L., Freccero M., Morales J.C. (2019). Carbohydrate-naphthalene diimide conjugates as potential antiparasitic drugs: Synthesis, evaluation and structure-activity studies. Eur. J. Med. Chem..

[27] Praliyev K.D., Yu V.K., Kanitar K., Muhidin A.O., Sundetova F., Kabdraissova A.Z. Design of new pharmocogically active aminopropargyles. Proceedings of the Abstracts of XIX Mendeleev Congress on General and Applied Chemistry.

[28] Kabdraissova A.Z., Faskhutdinov M.F., Yu V.K., Praliyev K.D., Fomichyova E.E., Shin S.N., Berlin K.D. (2007). Synthesis and properties of N-(2-ethoxyethyl)piperidine derivatives of anabasine. Chem. Nat. Compd..

[29] Kaldybayeva A.B., Malmakova A.Y., Li T.E., Ten A.Y., Seilkhanov T.M., Praliyev K.D., Yu V.K., Berlin K.D. (2022). Complexes of 3-[3-(1*H*-imidazol-1-yl)propyl]-3,7-diazabispidines and β-cyclodextrin as coatings to protect and stimulate sprouting wheat seeds. Molecules.

[30] Grella T.C., Soares-Lima H.M., Malaspina O., Cornelio Ferreira Nocelli R. (2019). Semi-quantitative analysis of morphological changes in bee tissues: A toxicological approach. Chemosphere.

[31] International Guiding Principles for Biomedical Research Involving Animals. Council for International Organization of Medical Sciences, Washington, DC, USA, 2012. https://media-01.imu.nl/storage/iclas.org/5196/cioms-iclas-principles-final.pdf.

[32] Kalra R., Singh S.P., Pena-Philippides J.C., Langley R.J., Razani-Boroujerdi S., Sopori M.L. (2004). Immunosuppressive and anti-inflammatory effects of nicotine administered by patch in an animal model. Clin. Diagn. Lam. Immunol..

[33] Sutunkove M.P., Ryabova Y.V., Minigalieva I.A., Bushueva T.V., Sakhautdinova R.R., Bereza I.A., Shaikhova D.R., Amromina A.M., Chemezov A.I., Shelomencev I.G. (2023). Features of the response to subchronic low-dose exposure to copper oxide nanoparticles in rats. Sci. Rep..

[34] Gurina T.S., Simms L. (2023). Histology, Staining. StatPearls. https://www.ncbi.nlm.nih.gov/books/NBK557663/#article-80132.s1.

