# Supplementary material for: Naphthaleneoxypropargyl-Containing Piperazine as a Regulator of Effector Immune Cell Populations upon an Aseptic Inflammation
Source: Molecules. 2023 Oct 11;28(20):7023. doi: 10.3390/molecules28207023 (PMC10608911; doi:10.3390/molecules28207023)
Supplement: Supplementary file 1 [file molecules-28-07023-s001.zip › molecules-2586174-supplementary.pdf]

Supporting information

Article

Naphthaleneoxypropargyl-Containing Piperazine as a Regulator of Effector Immune Cell Populations upon an Aseptic Inflammation

Valentina K. Yu [1,*], Yelena S. Sycheva [1], Gulgul K. Kairanbayeva [2], Valery M. Dembitsky [3,*], Marina K. Balabekova [2], Aliya N. Tokusheva [2], Tulegen M. Seilkhanov [4], Tolganay Y. Zharkynbek [1], Anar Kh. Balapanova [2] and Khaidar S. Tassibekov [1]

[1] A.B. Bekturov Institute of Chemical Sciences, 106 Sh. Ualikhanov St., Almaty 050010, Kazakhstan;
yelena-sycheva@yandex.kz (Y.S.S.); tolganay.zharkynbek@gmail.com (T.Y.Z.);
kh.tassibekov@ihn.kz (K.S.T.)
[2] Pathological Physiology Department, Asfendiyarov Kazakh National Medical University, 94 Tole-bi St., Almaty 050000, Kazakhstan; kairanbayeva.g@kaznmu.kz (G.K.K.);
balabekova.m@kaznmu.kz (M.K.B.);
aliyatokusheva88@gmail.com (A.N.T.); balapanova.an@kaznmu.kz (A.K.B.)
[3] Centre for Applied Research, Innovation and Entrepreneurship, Lethbridge College, 3000 College Drive South, Lethbridge, AB T1K 1L6, Canada
[4] Laboratory of Engineering Profile NMR Spectroscopy, Sh. Ualikhanov Kokshetau State University,
76 Abai St., Kokshetau 020000, Kazakhstan; tseilkhanov@mail.ru
* Correspondence: yuvkconst@gmail.com (V.K.Y.); valery.dembitsky@lethbridgecollege.ca or valery@dembitsky.com (V.M.D.)

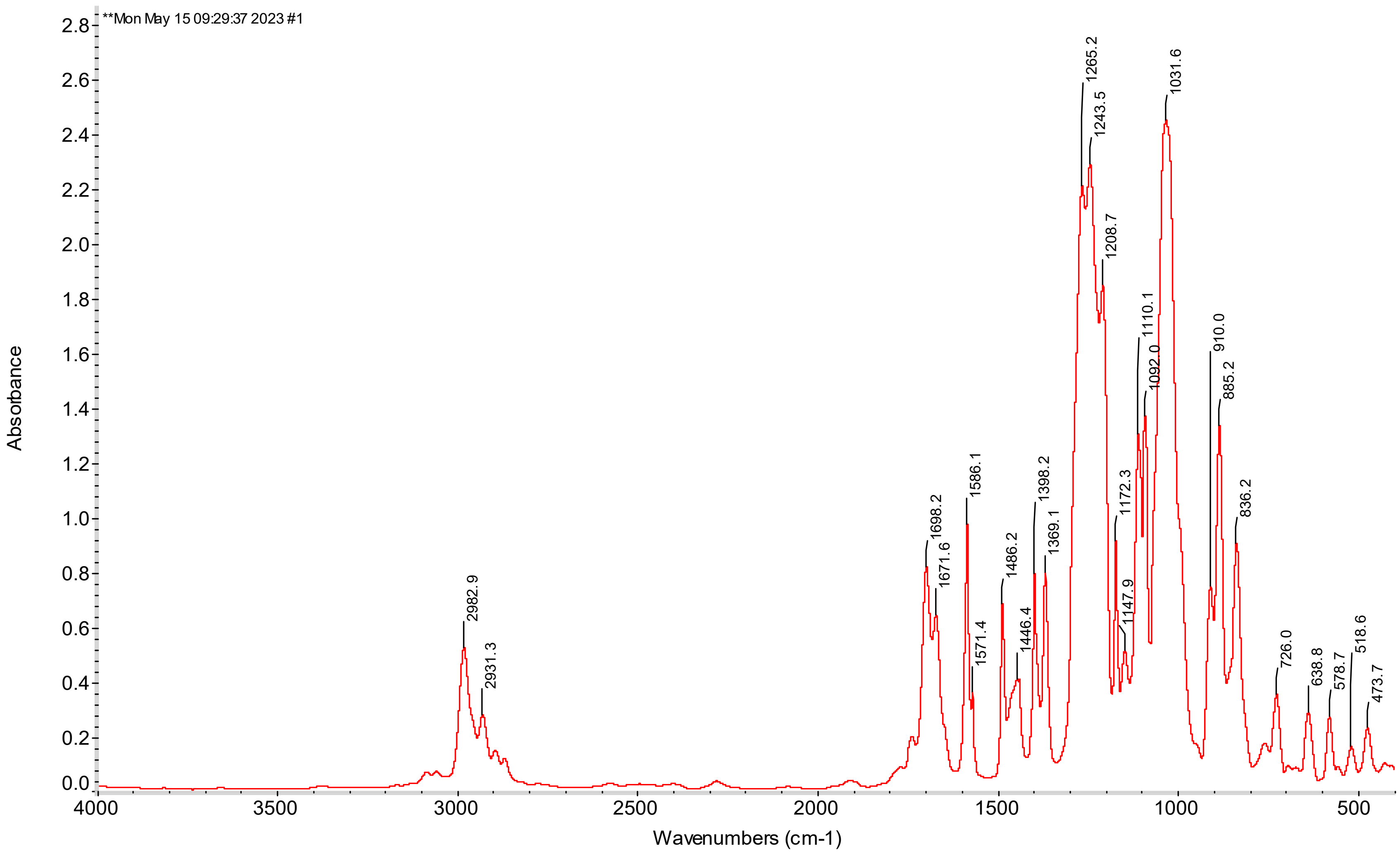

Figure S1. IR (KBr, ν, cm^{-1}) spectrum of 1-[1-(2,5-dimethoxyphenyl)-4 (naphthalene-1-yloxy)but-2-ynyl]-4-methylpiperazine.

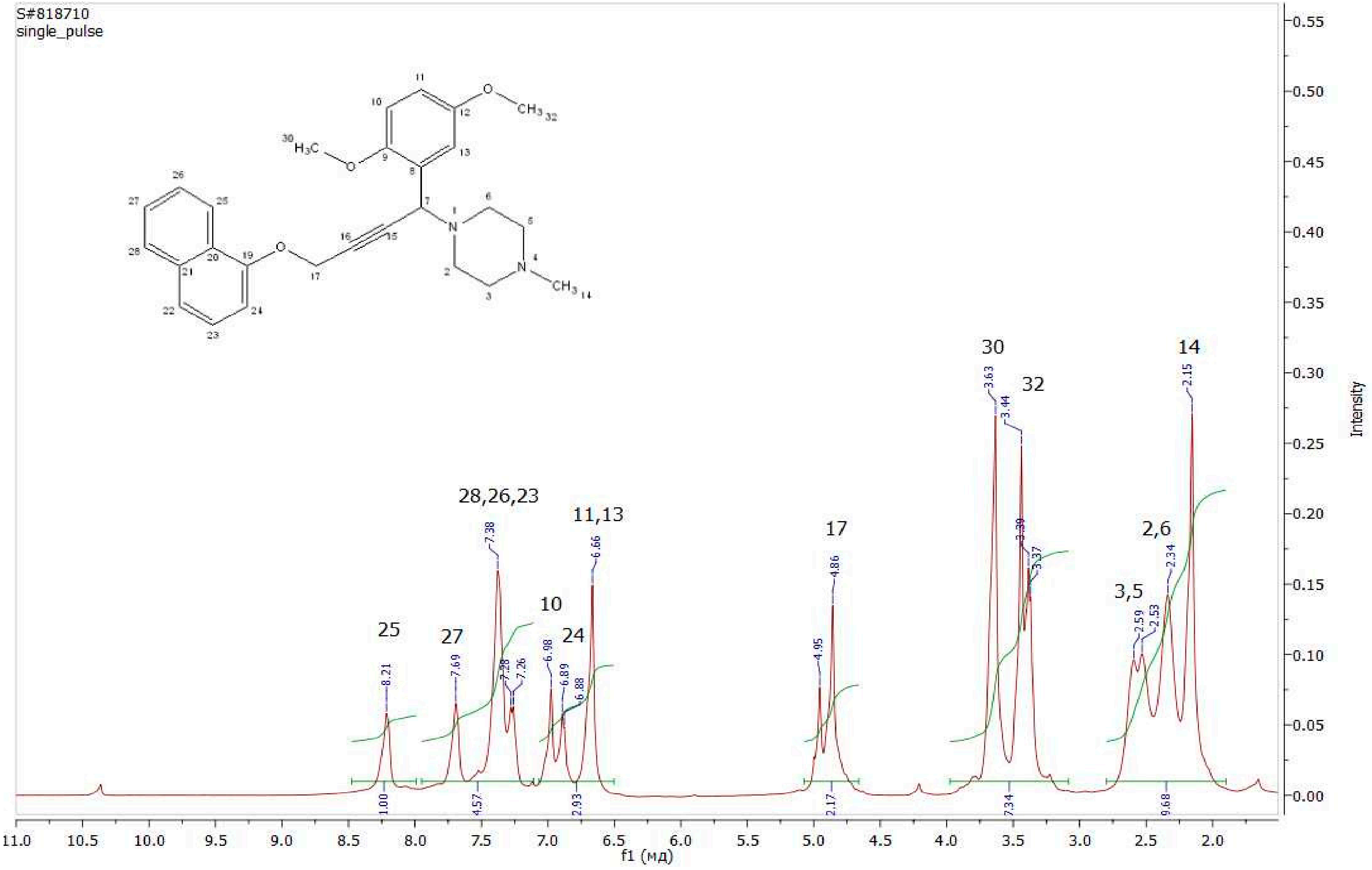

Figure S2. ^{1}H NMR (399.78 MHz, $CDCl_3$) spectrum of 1-[1-(2,5-dimethoxyphenyl)-4-(naphthalene-1-yloxy)but-2-ynyl]-4-methylpiperazine.

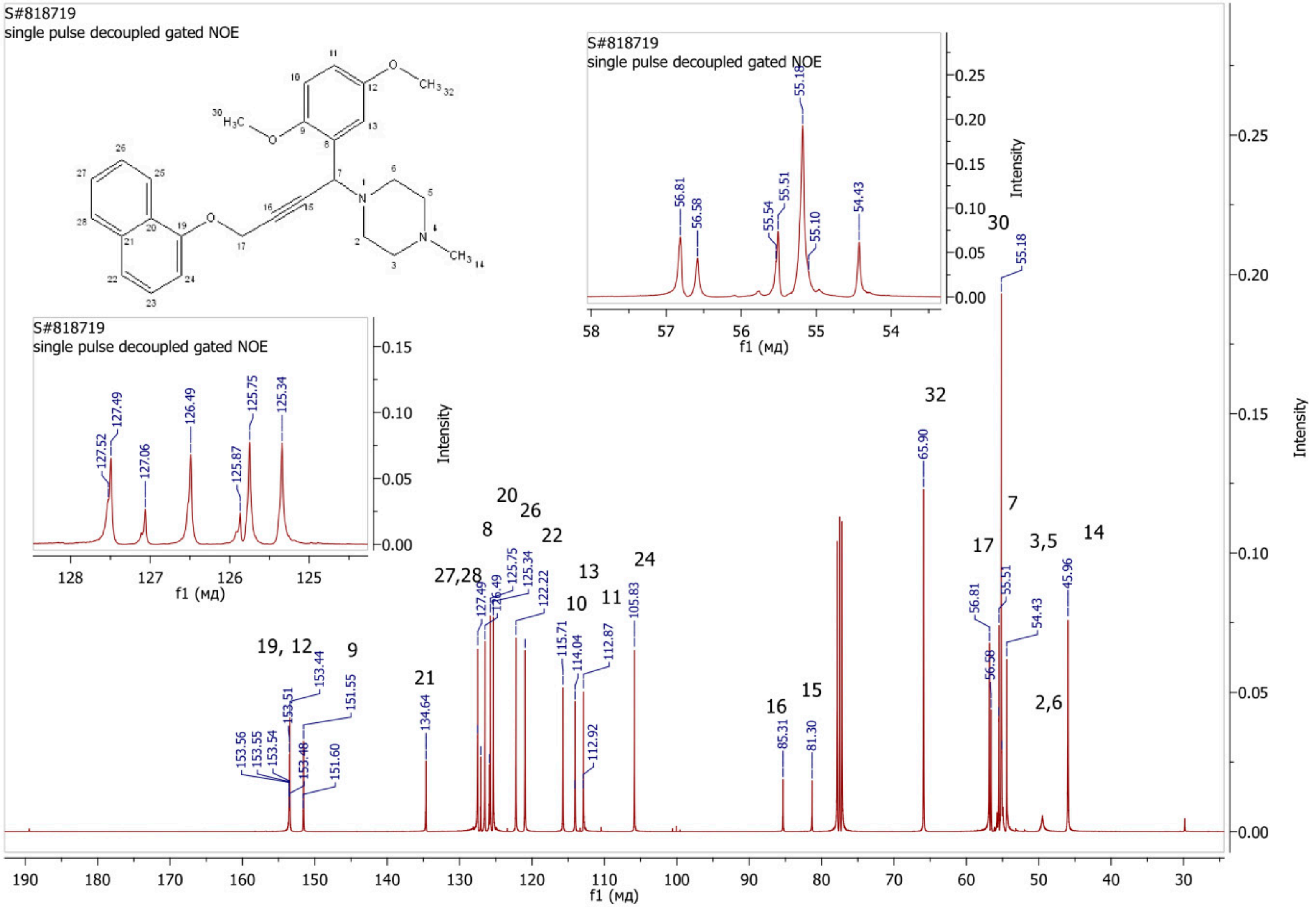

Figure S3. ^{13}C NMR (100.53 MHz, $CDCl_3$) spectrum of 1-[1-(2,5-dimethoxyphenyl)-4-(naphthalene-1-yloxy)but-2-ynyl]-4-methylpiperazine.

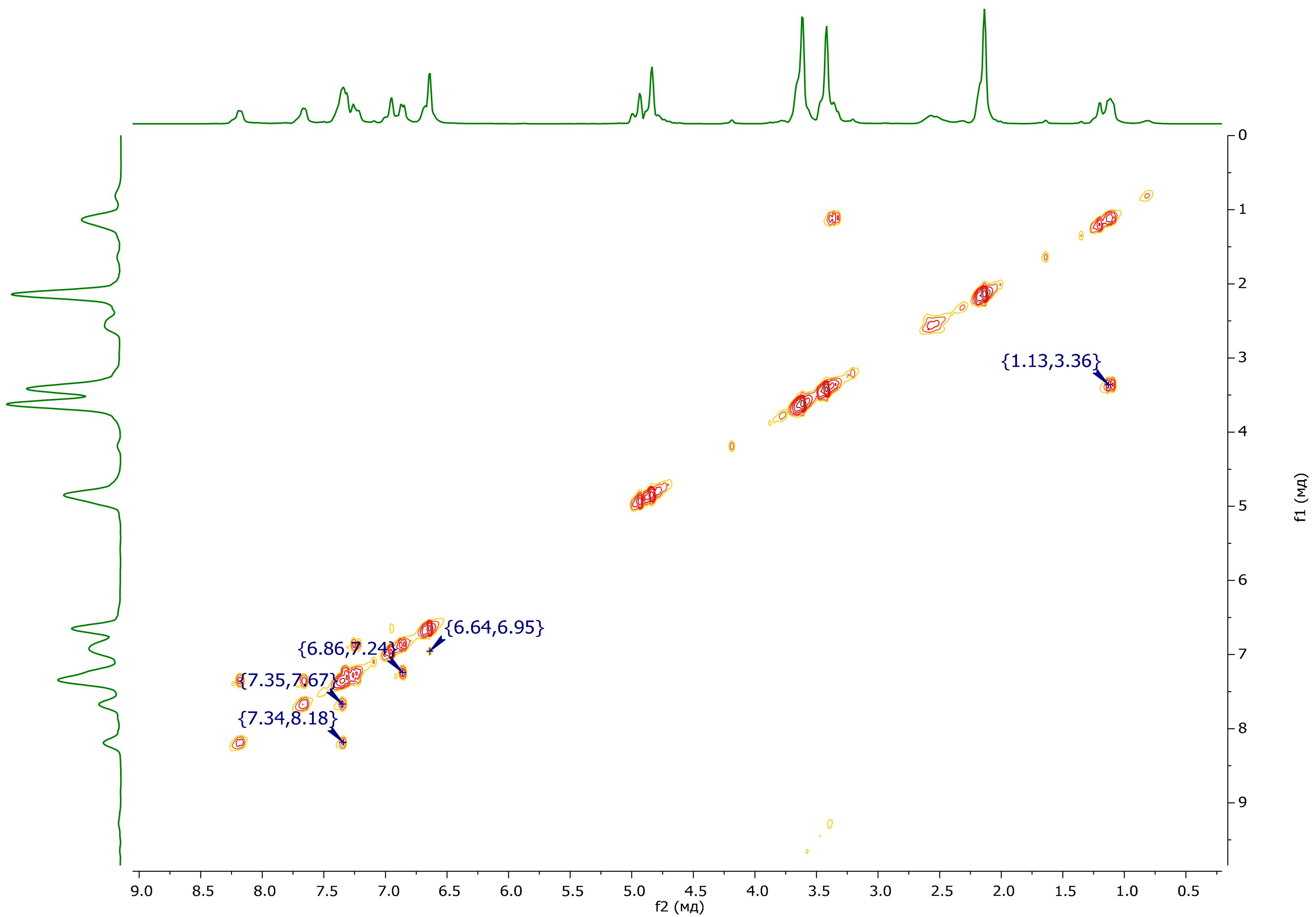

Figure S4. ^{1}H-^{1}H COSY spectrum of 1-[1-(2,5-dimethoxyphenyl)-4-(naphthalene-1-yloxy)but-2-ynyl]-4-methylpiperazine.

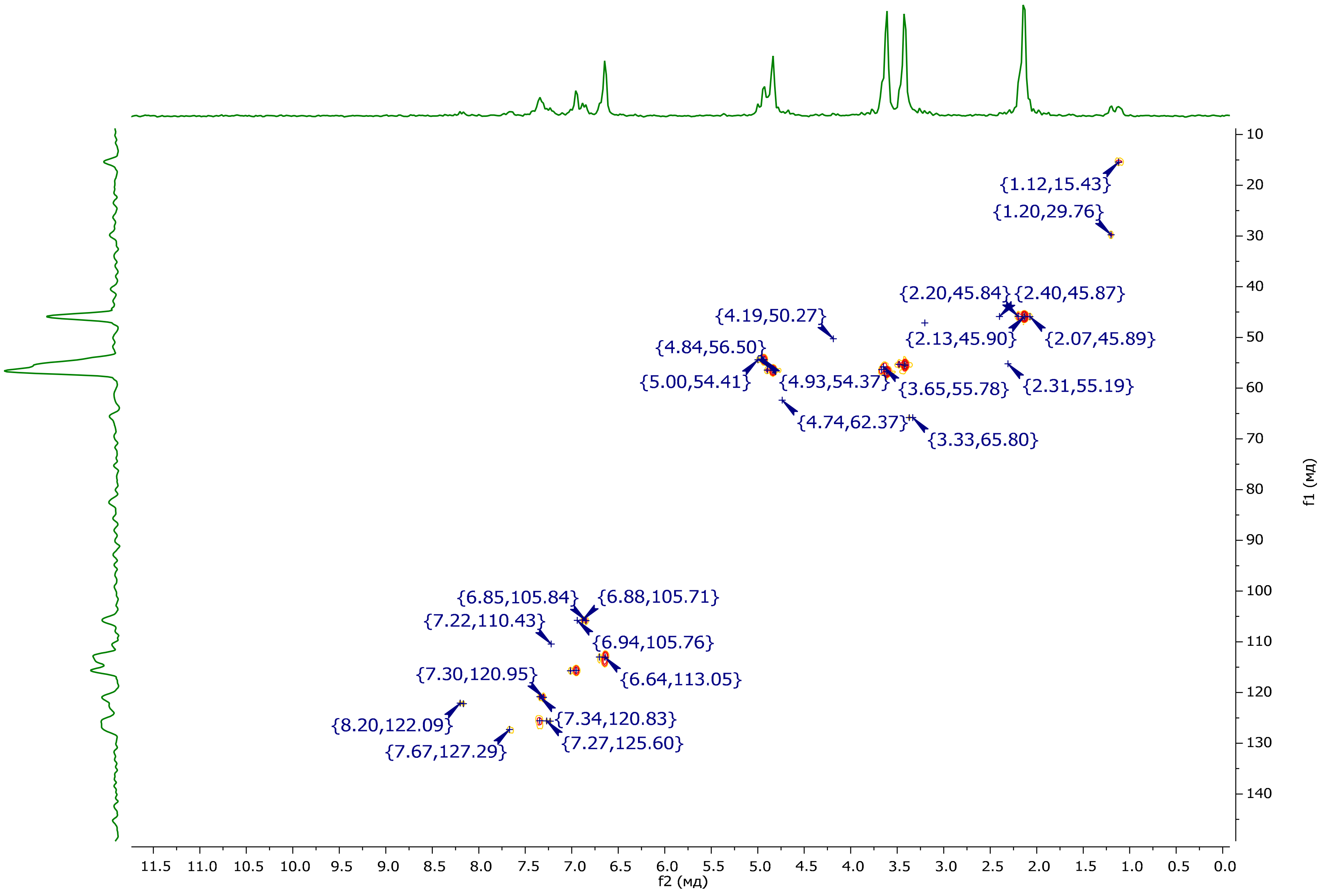

Figure S5. ^{1}H-^{13}C HMQC spectrum of 1-[1-(2,5-dimethoxyphenyl)-4-(naphthalene-1-yloxy)but-2-ynyl]-4-methylpiperazine.

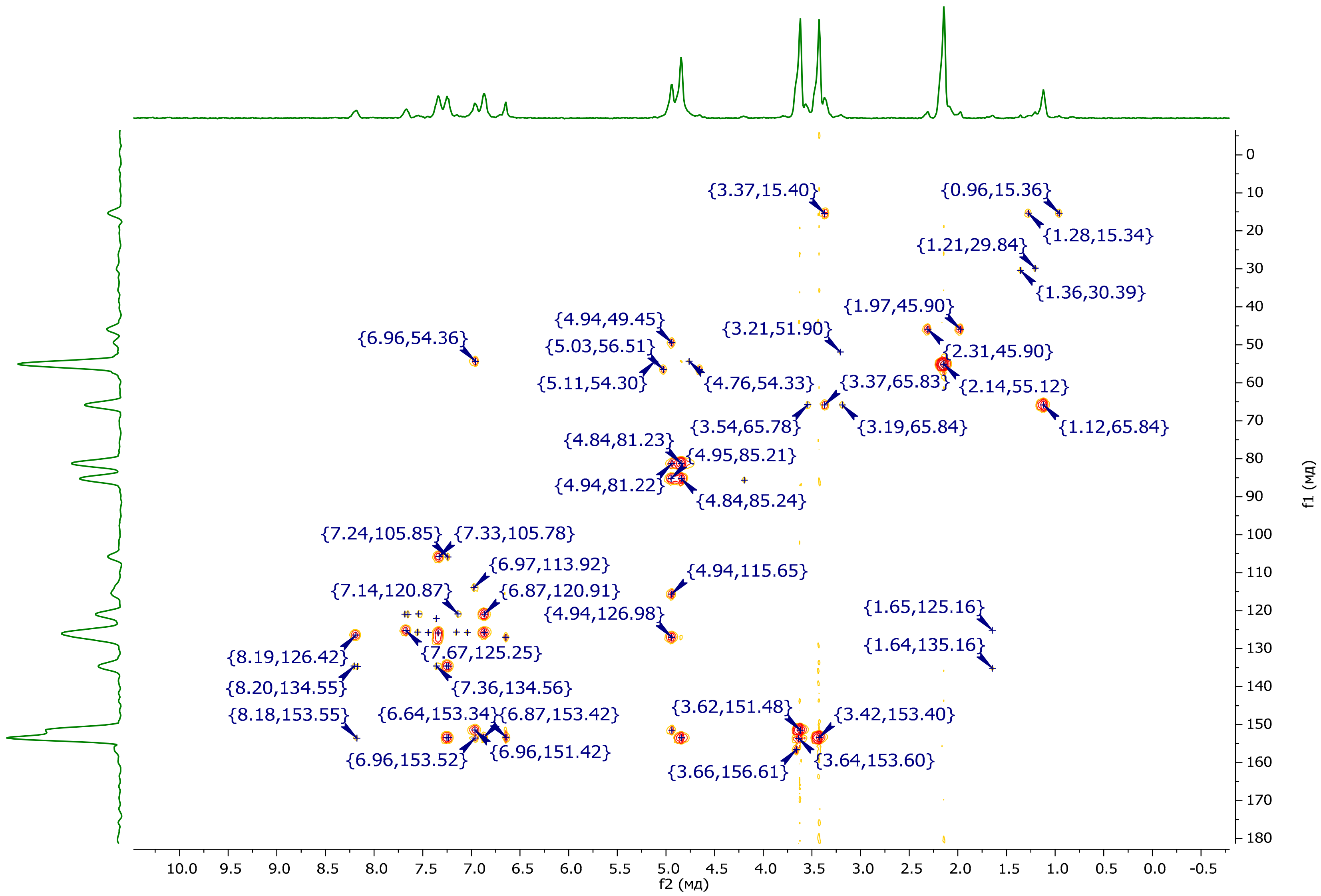

Figure S6. ^{1}H-^{13}C HMBC spectrum of 1-[1-(2,5-dimethoxyphenyl)-4-(naphthalene-1-yloxy)but-2-ynyl]-4-methylpiperazine.

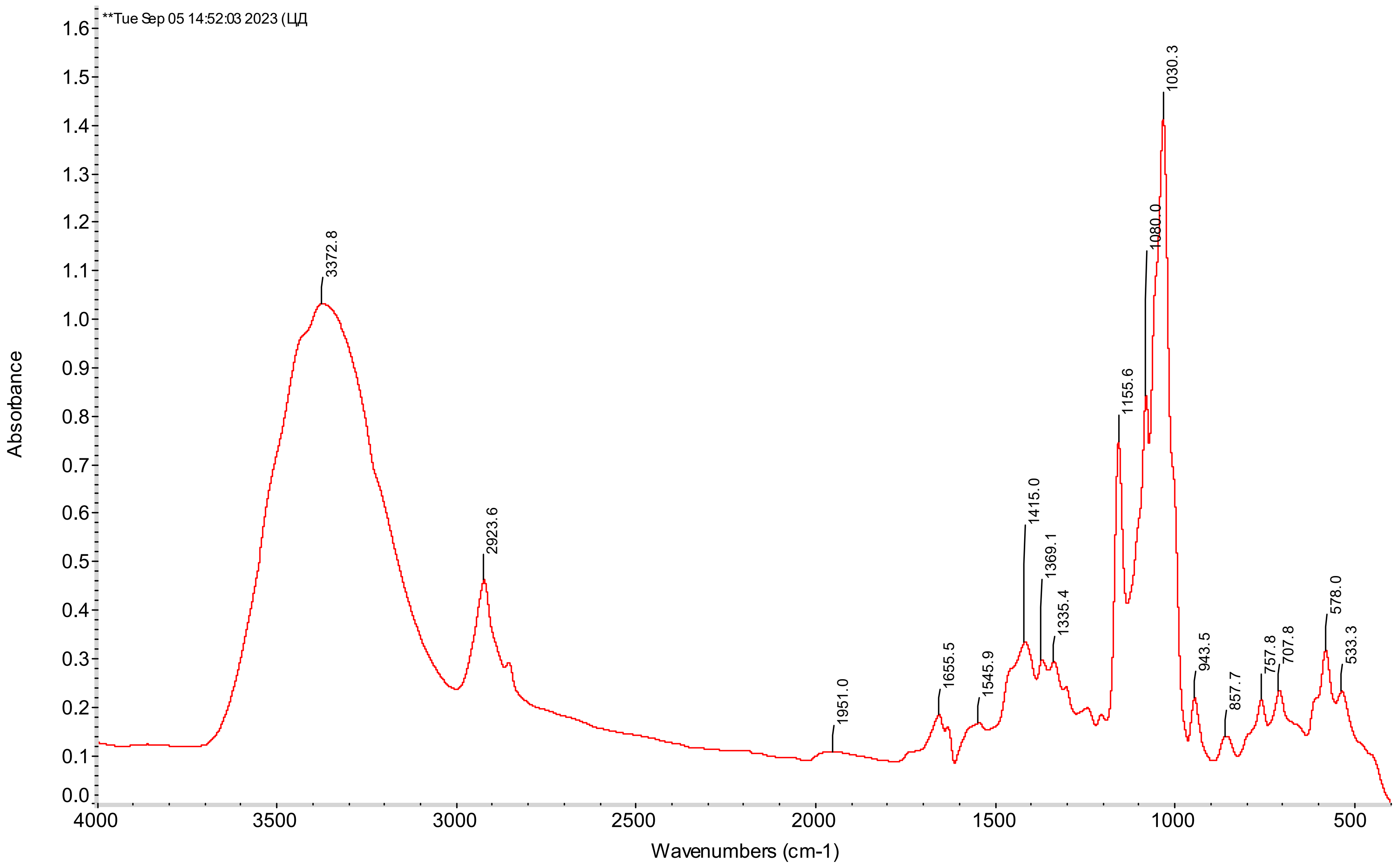

Figure S7. IR (KBr, ν, cm^{-1}) spectrum of β-cyclodextrin (β-CD).

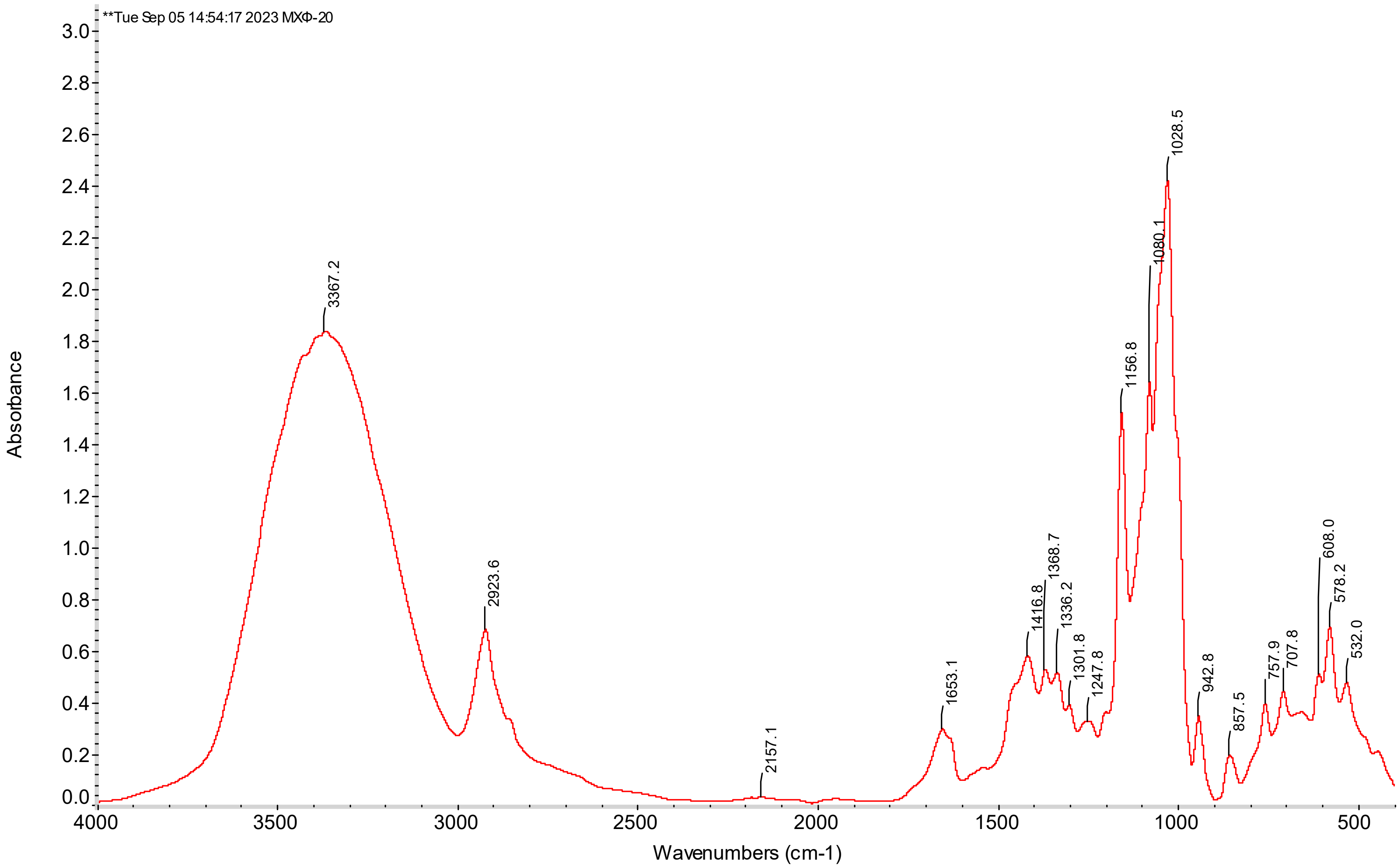

Figure S8. IR (KBr, ν, cm⁻¹) spectrum of 1-[1-(2,5-dimethoxyphenyl)-4-(naphthalene-1-yloxy)but-2-ynyl]-4-methylpiperazine with β-cyclodextrin (***complex***).

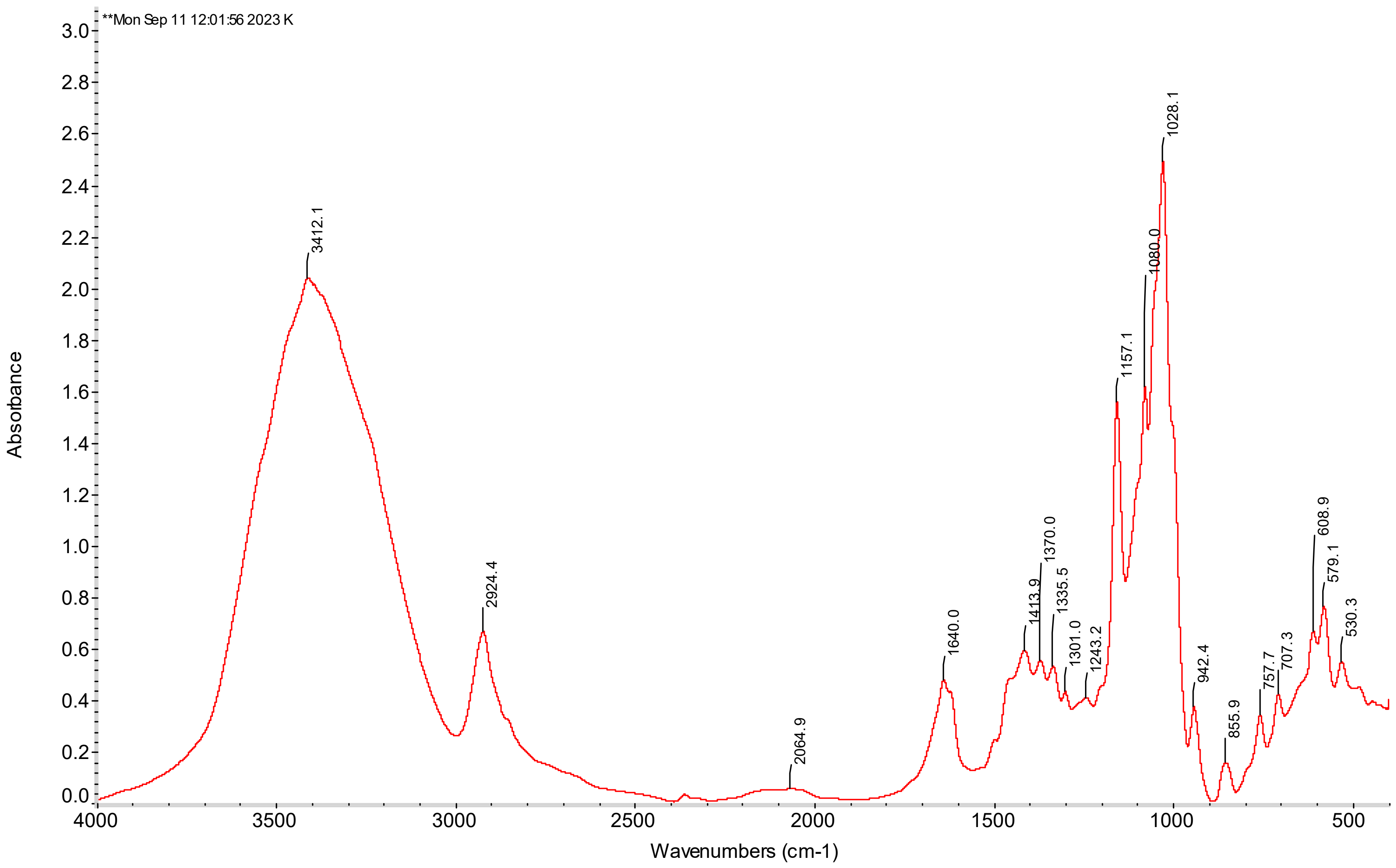

Figure S9. IR (KBr, ν, cm^{-1}) spectrum of 1-[1-(2,5-dimethoxyphenyl)-4-(naphthalene-1-yloxy)but-2-ynyl]-4-methylpiperazine with β-cyclodextrin (physical mixture)
